# Supplementary material for: MiRNA-Mediated Fibrosis in the Out-of-Target Heart following Partial-Body Irradiation
Source: Cancers (Basel). 2022 Jul 16;14(14):3463. doi: 10.3390/cancers14143463 (PMC9323333; doi:10.3390/cancers14143463)
Supplement: Supplementary file 1 [file cancers-14-03463-s001.zip › Supplementary Figure S1.pptx]

## Slide 1
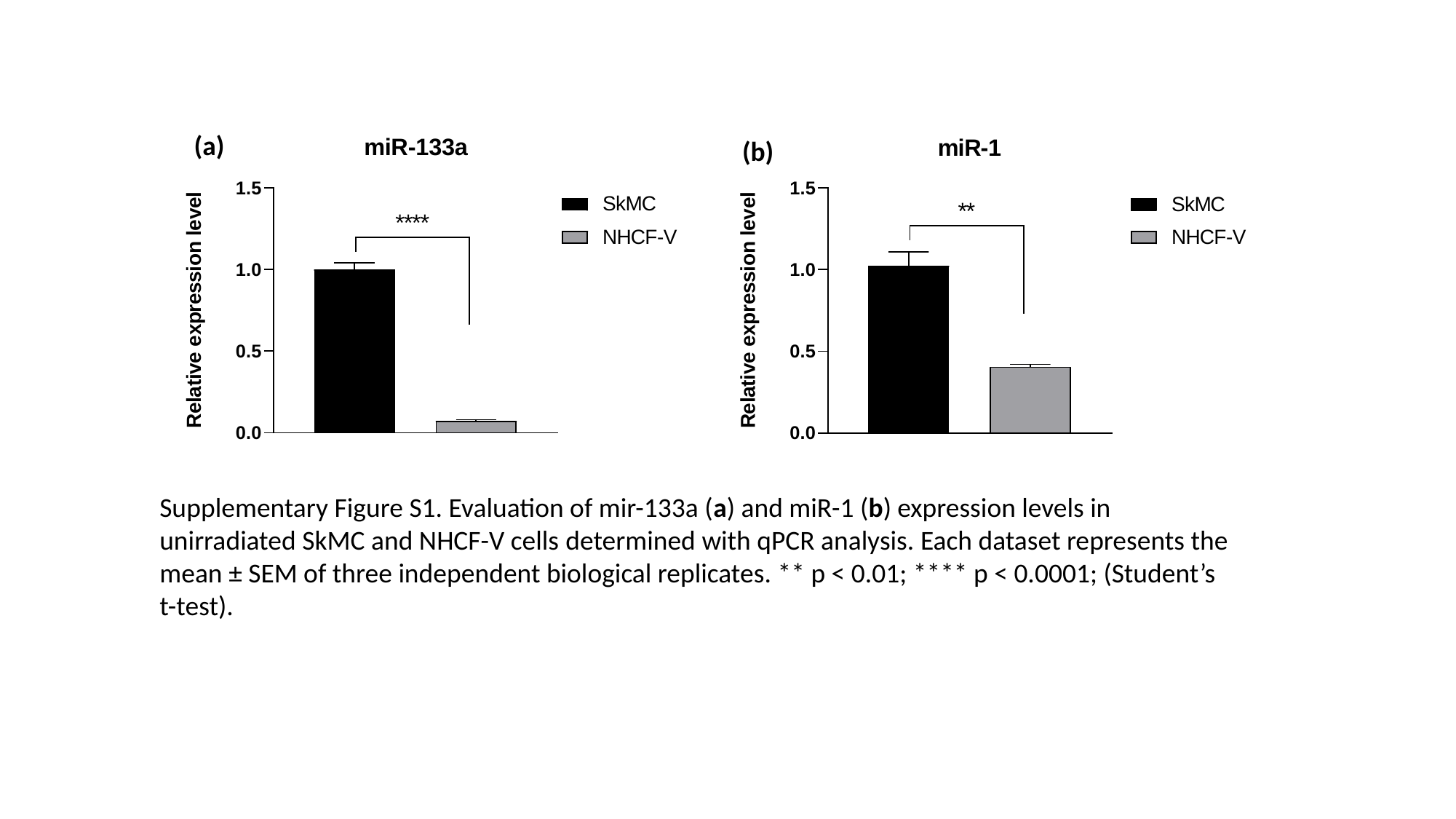

(a)
(b)
Supplementary Figure S1. Evaluation of mir-133a (a) and miR-1 (b) expression levels in unirradiated SkMC and NHCF-V cells determined with qPCR analysis. Each dataset represents the mean ± SEM of three independent biological replicates. ** p < 0.01; **** p < 0.0001; (Student’s t-test).
