# Supplementary material for: MiRNA-Mediated Fibrosis in the Out-of-Target Heart following Partial-Body Irradiation
Source: Cancers (Basel). 2022 Jul 16;14(14):3463. doi: 10.3390/cancers14143463 (PMC9323333; doi:10.3390/cancers14143463)
Supplement: Supplementary file 1 [file cancers-14-03463-s001.zip › Supplementary Table S1.pdf]

Supplementary Table S1. List of primers used for quantitative real time PCR.

Gene	Forward primer	Reverse primer	Species
Vim	5'- GAGAGAGGAAGCCGAAAGCA -3'	5'- GCCAGAGAAGCATTGTCAACATC -3'	Mouse
Fn1	5'- CGAGGTGACAGAGACCACAA -3'	5'- CTGGAGTCAAGCCAGACACA -3'	Mouse
α-SMA	5'- GAGGCACCACTGAACCCTAA -3'	5'- CATCTCCAGAGTCCAGCACA -3'	Mouse
Col3a1	5'- TCCCCTGGAATCTGTGAATC -3'	5'- TGAGTCGAATTGGGGAGAAT -3'	Mouse
Gadph	5' - CATGGCCTTCCGTGTTCCTA -3'	5'- GCGGCACGTCAGATCGA -3'	Mouse
FN1	5'- CGAGGTGACAGAGACCACAA -3'	5'- CTGGAGTCAAGCCAGACACA -3'	Human
COL1A1	5'- GCTACCCAACTTGCCTTCATG -3'	5'- GCTGTTCTTGCAGTGGTAGGTG -3'	Human
COL3A1	5'- CCCACTATTATTTTGGCACAACAG -3'	5'- GCATGGTTCTGGCTTCCAGA -3'	Human
CTGF	5'- ATTAGAGCCAACTGCCTGGT -3'	5'- AGGAGGCGTTGTCATTGGTA -3'	Human
GADPH	5' - ATTCCACCCATGGCAAATTC -3'	5'- GGGATTTCCATTGATGACA -3'	Human
